# Highly diverse Bronze Age population dynamics in Central-Southern Europe and their response to regional climatic patterns

**DOI:** 10.1371/journal.pone.0200709

**Published:** 2018-08-08

**Authors:** Giacomo Capuzzo, Marco Zanon, Marta Dal Corso, Wiebke Kirleis, Juan A. Barceló

**Affiliations:** 1 Laboratory of Anthropology and Human Genetics, Faculty of Science, Université Libre de Bruxelles, Brussels, Belgium; 2 Quantitative Archaeology Lab (LAQU), Department of Prehistory, Autonomous University of Barcelona, Faculty of Arts and Humanities, Bellaterra (Barcelona), Spain; 3 Graduate School “Human Development in Landscapes”, Kiel University, Kiel, Germany; 4 Institute of Pre- and Protohistoric Archaeology, Kiel University, Kiel, Germany; New York State Museum, UNITED STATES

## Abstract

The reconstruction of past demographic patterns is a fundamental step towards a better understanding of human-environment relations, especially in terms of quantifiable anthropic impact and population susceptibility to environmental changes. The recently developed Summed Calibrated Probability Distributions (SCPD) approach, based on large collections of archaeological radiocarbon dates, provides a new tool to obtain continuous prehistoric population curves suitable for comparison with palaeoenvironmental time series. Despite a wide application in Mesolithic and Neolithic contexts worldwide, the use of the SCPD method remains rare for post-Neolithic societies. Our aim is to address this visible gap and apply the SCPD approach to South European archeological contexts between the Bronze Age and the transition into the Iron Age (1800–800 cal. BC), then evaluating these results against local archeological narratives and palaeoecological data. We first test the SCPD method at a supra regional scale, ranging from the Ebro to the Danube rivers, and subsequently in five selected regions within this area. We then compare the regional population curves to climate data reconstructed from local palynological records. Our results highlight the contrast between a stable supra regional demographic trend and more dynamic regional patterns. We do not observe any convincing long-term correlations between population and climate, but localized episodes of demographic stagnation or decline are present in conjunction with climatic shifts or extremes. Nevertheless, climate change as a triggering factor should be considered with caution, especially in peripheral areas where the archaeological data is faint, or where local evidence points to contemporaneous, ongoing landscape overexploitation.

## 1. Introduction

The reconstruction of past population size and density has always been a major challenge for archaeological research. Early attempts were based on the analysis of settlement sizes and funerary contexts, leading to the hypothesis that there was a significant population increase over a large part of Europe during the Late Bronze Age [1–3]. One well-known case study cites the rise in the number of barrows and an increase in burial wealth at around 1200 BC in Northern Europe, and a visible increase in the number of necropolises in Poland from 1500–1400 BC [1,4]. Archaeologists also observed a peak in the number of lakeside settlements in Switzerland during the same period [5]. The hypothesis that there was a population increase during the Late Bronze Age [1] was strengthened further by geostatistical analysis of site densities in the landscape [6,7]. Yet, the European Bronze Age was also characterized by episodes of crisis, leading to the end of some settlement systems and to phases of widespread depopulation [8]. A major example in our study area is the end of the lake-dwelling settlement system in the Circum-Alpine region. The pile-dwelling phenomenon expanded to its maximum during the first phases of the Bronze Age, covering a wide area ranging from Eastern France to Slovenia [9,10]. An abrupt abandonment phase is then recorded north of the Alps in partial connection with high lake level stands (1400–1150 BC). Conversely, the settlement network south of the Alps was relatively stable through most of the Bronze Age, eventually peaking in population density with the development of the Terramare culture in the neighboring Po Plain [11,12]. The southern Alpine pile-dwelling/Terramare culture collapsed around 1150 BC, probably due to a combination of factors including unsustainable landscape exploitation and climatic change [13–15].

In recent years, the creation of large databases of radiocarbon dates has enabled the use of SCPDs to infer relative population dynamics. The number of datable archeological contexts can be a direct function of human pressure on the landscape (e.g. [16]). Consequently, frequency fluctuations in a series of SCPDs can be interpreted as a reflection of past population trends. Archaeologists have given particular attention to the Neolithic period [16–24] using SCPDs to track population dynamics in connection with the spread of agricultural practices. However, this methodology has been rarely applied to the so-called ‘metal ages’. Notable examples for the European Bronze Age appear to be limited to the works of Armit et al. [25,26], focusing on the reconstruction of Irish population trends in connection with the Subboreal-Subatlantic transition, and Balsera et al. [27] on the demography of prehistoric Iberia from 7000 to 2000 BC.

**Fig 1:**
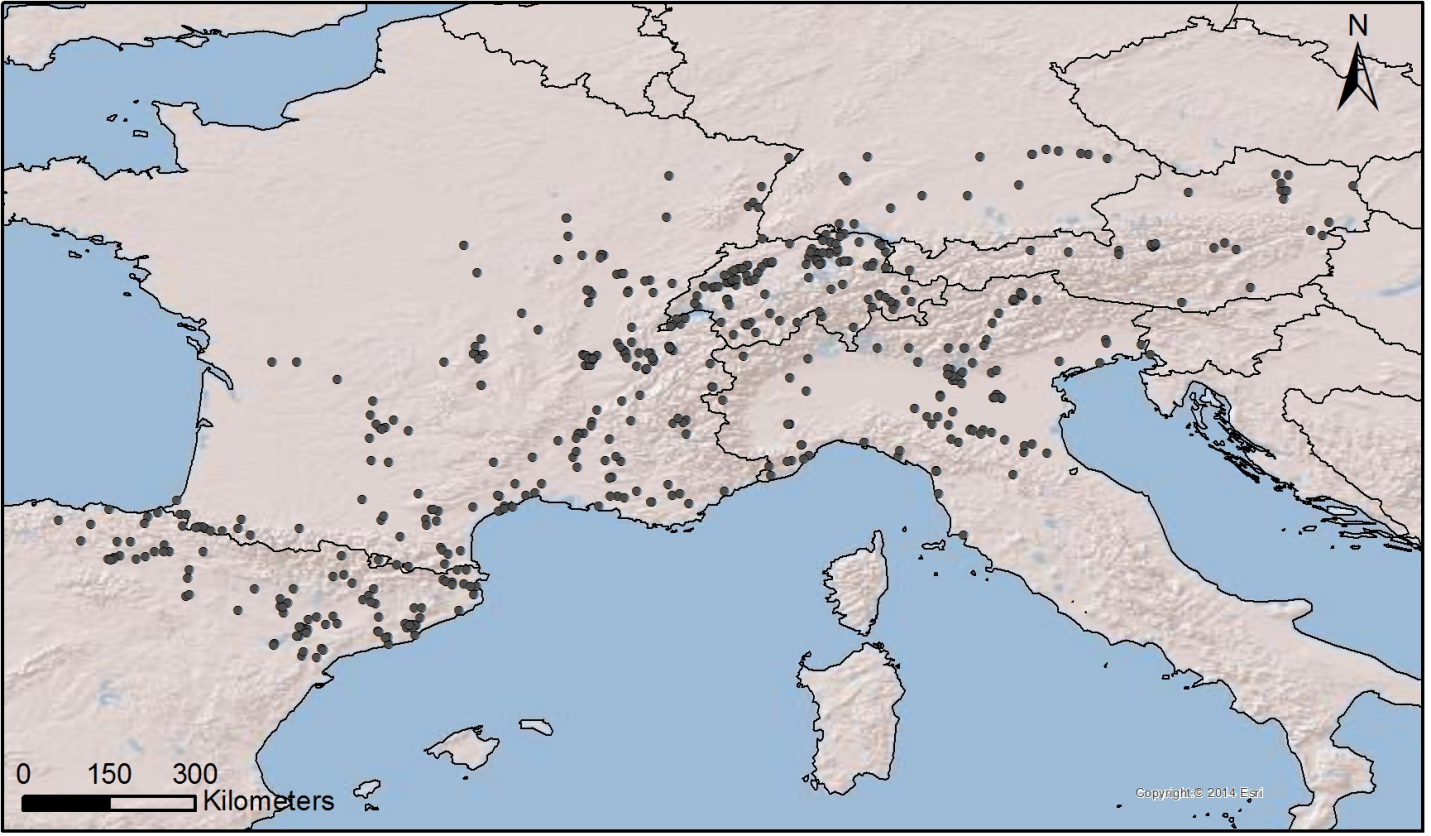
Overview of the study area. The black dots show the spatial distribution of the archaeological sites included in the EUBAR database and used to derive population data (Software: ArcGIS10.3).

In the present paper, we use the SCPD method to track human activity from the Ebro to the Danube Rivers, including the Northeastern Iberian Peninsula, Central and Southern France, Northern Italy, Switzerland, Austria, and Southern Germany (Fig 1). We focus our reconstructions on 1800–800 BC, broadly covering most of the Bronze Age and the transition into the Iron Age.

**Fig 2:**
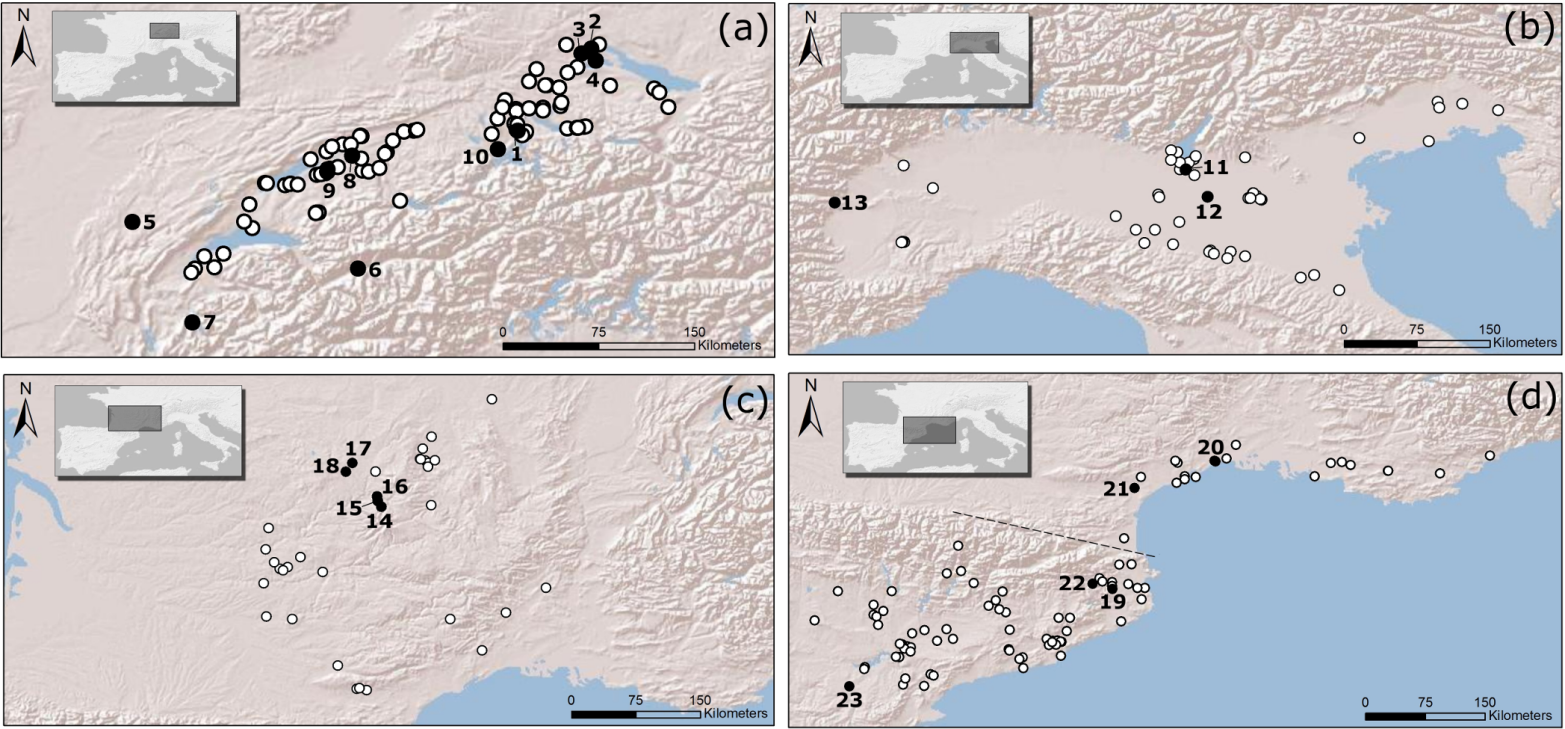
Regions investigated in the present study: (a) Swiss Plateau; (b) Po Plain; (c) Massif Central; (d) Southern French coast and Northeastern Iberian Peninsula. The latter two regions are also jointly referred to as ‘Northwestern Mediterranean’ in the present paper. White dots: sites included in the regional SCPDs, selected from the EUBAR dataset. Black dots: sites used for climatic reconstructions (coordinates reported in Table 1). (1) Bibersee; (2) Durchenbergried; (3) Feuenried; (4) Hornstaad/Bodensee; (5) Lac de Clairvaux; (6) Lac du Mont d'Orge; (7) Lac d’Annecy; (8) Lobsigensee; (9) Montilier; (10) Rotsee; (11) Castellaro Lagusello; (12) Forcello; (13) Lago Piccolo di Avigliana; (14) La Taphanel; (15) Lac du Mont de Belier; (16) Lastioulles; (17) Peyrelevade; (18) Tourbière de Chabannes; (19) Banyoles; (20) Embouchac; (21) Etang d'Ouveillan; (22) Pla de l`Estany; (23) Salada Pequeña.

This larger area is subsequently divided into five regions (the Swiss Plateau, the Po Plain, the Massif Central, the Southern French coast, and the Northeastern Iberian Peninsula, Fig 2) in order to track more specific trends and inter-regional dynamics.

The regional demographic trends are then compared with semi-quantitative climatic curves reconstructed from local pollen archives. We use the Modern Analogue Technique (MAT; [28,29]) to reconstruct summer temperature and precipitation from different pollen archives selected in the five regions and their immediate vicinities. The temperature and precipitation reconstructions for each group of sites within a region are then combined into two respective LOESS-smoothed curves. The resulting highly localized synthesis offered by these two climatic parameters allows for a geographically consistent comparison with the demographic data. The continuous, semi-quantitative, and localized nature of these reconstructions offer an independent testing ground for established regional palaeoenvironmental narratives based on combinations of discrete, qualitative, and extra-regional data.

## 2. Materials and methods

### 2.1. Demographic reconstructions

Several techniques have been used to estimate the probable size of a human population based on archaeological data, such as the study of settlement size, house dimensions, and site catchment areas, as well as the measurement of the rates of exploitation, consumption, and discard of raw materials and artifacts [30]. Similarly, the analysis of funerary contexts and human skeletons has been used to estimate past population size through the inference of age-specific mortality from assemblages of human skeletal remains [31]. A recent tool developed in this field focuses on the use of summed calibrated probability distributions (SCPD) of radiocarbon dates from archaeological sites in order to track trends in human presence. The originality of calibrating ^14^C data to infer population structure (and use the pooled mean of the “dates as data” to do so) comes from K. Edinborough’s 2005 PhD thesis [32,33]. The SCPD method is based on the reasonable assumption that as the number of people increases, so does the strength of their archaeological signal. Consequently, changes in the relative temporal frequency of radiocarbon dated depositional events are interpreted as reflections of demographic trends.

In the present paper, we adopt the SCPD method to infer past demographic fluctuations during the Bronze Age and towards the transition into the Iron Age.

We produced the general South European demographic reconstruction by combining 1233 calibrated radiocarbon dates selected from the EUBAR database [34], openly accessible online at http://www.telearchaeology.org/EUBAR and at https://figshare.com/s/be0d5df7df1fff96f7bb. Subsequently, we composed separate SCPD curves for each one of the selected regions using subsections of the EUBAR dataset. All related analyses have been carried out through the software OxCal 4.3 [35] using the IntCal13 calibration curve [36].

The SCPD approach has been the target of several criticisms (e.g. [37–40]). Interpretative and methodological difficulties in the SCPDs can be divided in three groups: those referring to the systematic bias in the available data, mainly due to different ranges of preservation and to sampling strategies [26,41], those linked to the artificial results generated by the graphical methods of presenting the data [42] and those related to the effect of the calibration process [40,43,44]. Nonetheless, its wide adoption and testing within several archaeological contexts support its ability to infer past human dynamics [16,20,25–27,41,45–51]. In this framework, the EUROEVOL project led by Stephen Shennan at UCL (http://www.ucl.ac.uk/euroevol) deserves to be mentioned for its relevant outcomes [19–20].

Summing a group of estimates that have different probabilities produces a unique probability density function for a hypothetically defined period, which is the sum of the individual confidence intervals of the radiocarbon dates. Observed positive trends in the SCPD may be interpreted as a sign of increasing population, while decreasing SCPD values would point to demographic declines. Consequently, the steepness of the slope may indicate the speed of increase or decrease.

In the present paper, we combined radiocarbon dates from the same depositional event in order to prevent the non-independence of dated events [52,53]. In so doing, we have followed a pre-analytic “binning” procedure, instead of the more usual post-analysis “binning” described in recent contributions [16,20,43,48,54–57]. We also adopted a range of pre-screening criteria in order to ensure the reliability of our analysis. First, only radiocarbon measurements with a standard deviation of less than 95 years have been taken into account, allowing a reduction in global uncertainty while at the same time maintaining a reasonable sample size. Adopting a stricter cutoff for standard deviations (e.g. 40/50 years) would result in a loss of information, excluding reliable although less precise dates. In this regard, it is worth quoting Shennan (p. 305 in [19]), who maintains that “(the) key point is that even though a single date may have a broad calibrated range, the accumulation of the probability distributions of a large number of dates produces a high degree of chronological resolution making it possible to trace population fluctuations in considerable detail”.

Second, we attempted to guarantee an equal representation between sites with only a few radiocarbon dates and multi-dated archaeological contexts. Dates from the same archaeological context and corresponding to the same depositional event (for instance two bones from the same individual in a grave) were tested following Ward and Wilson [58]. When the test was positive, the uncalibrated dates were combined using the tool *R_Combine* of the program OxCal 4.3, then their pooled mean was calibrated [35]. In this way, when summing the radiocarbon estimates resulting from both combined contexts and mono-dated layers, the precision and the accuracy of the SCPDs remarkably improve and the representation of archaeological contexts is not altered.

To understand the magnitude of the prescreening criteria adopted in the paper it is meaningful to observe that, on a sample of 1785 radiocarbon dates which compose the EUBAR database, only 852 dates from settlements were retained to construct the SCPD adopted to infer demographic changes at a macro scale. When analyzing the geographical regions separately, we are limited to datasets of less than 500 dates. Although this may be a problem when analyzing very long temporal ranges (more than 5000 years) [40], it is less problematic in shorter temporal ranges -as the 1000 years here studied- and when data density per year and per square kilometer are above critical thresholds. See section 4 (discussion) for additional considerations on data density.

On a macroscale, radiocarbon dates from cemeteries and from settlements have been analyzed separately. However, due to the small amount of data at a regional scale, the dates from both contexts have been considered jointly.

**Fig 3:**
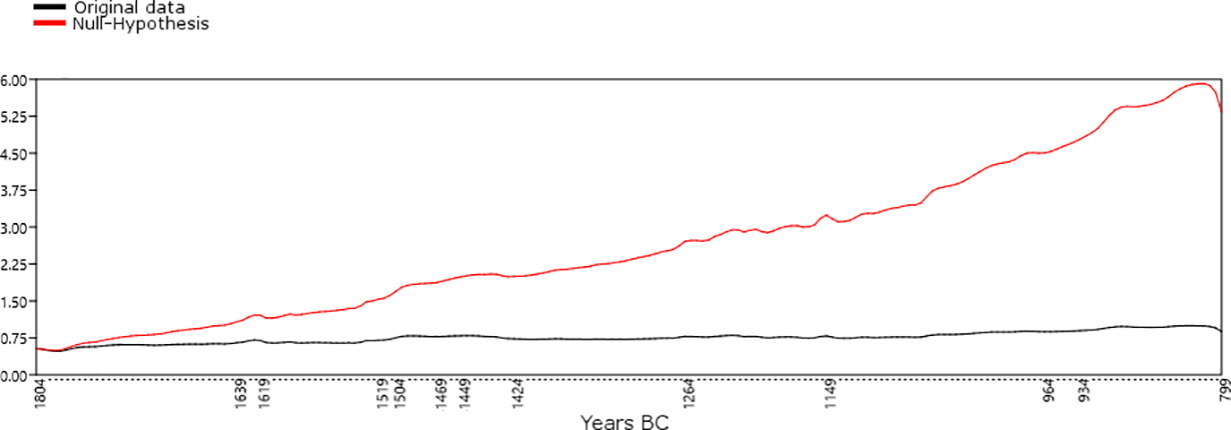
Testing the effect of taphonomic bias on the original SCPD from settlements (Software: PAST 3.18). Black line: Original data; red line: Null-Hypothesis.

In order to evaluate whether taphonomic bias may affect the general trend of the temporal series, we followed the approach of Surovell et al. [38] and tested our data against a null-hypothesis based on the equation *n_t_* = 5.726442 × 10^6^(*t* + 2176.4)^−1.3925309^, where *t* has been defined for the interval 1800-800 BC. In Fig 3, the original SCPD data for the period 1800-800 BC (in black) are plotted against a curve derived from the null-hypothesis equation *n_t_* (in red), built upon the assumption that post-depositional bias explains the higher amount of recent ^14^C samples in terms of higher occurrences of better preserved contexts. The widely different trends of the two curves suggest that our data are not significantly affected by taphonomic bias.

**Fig 4:**
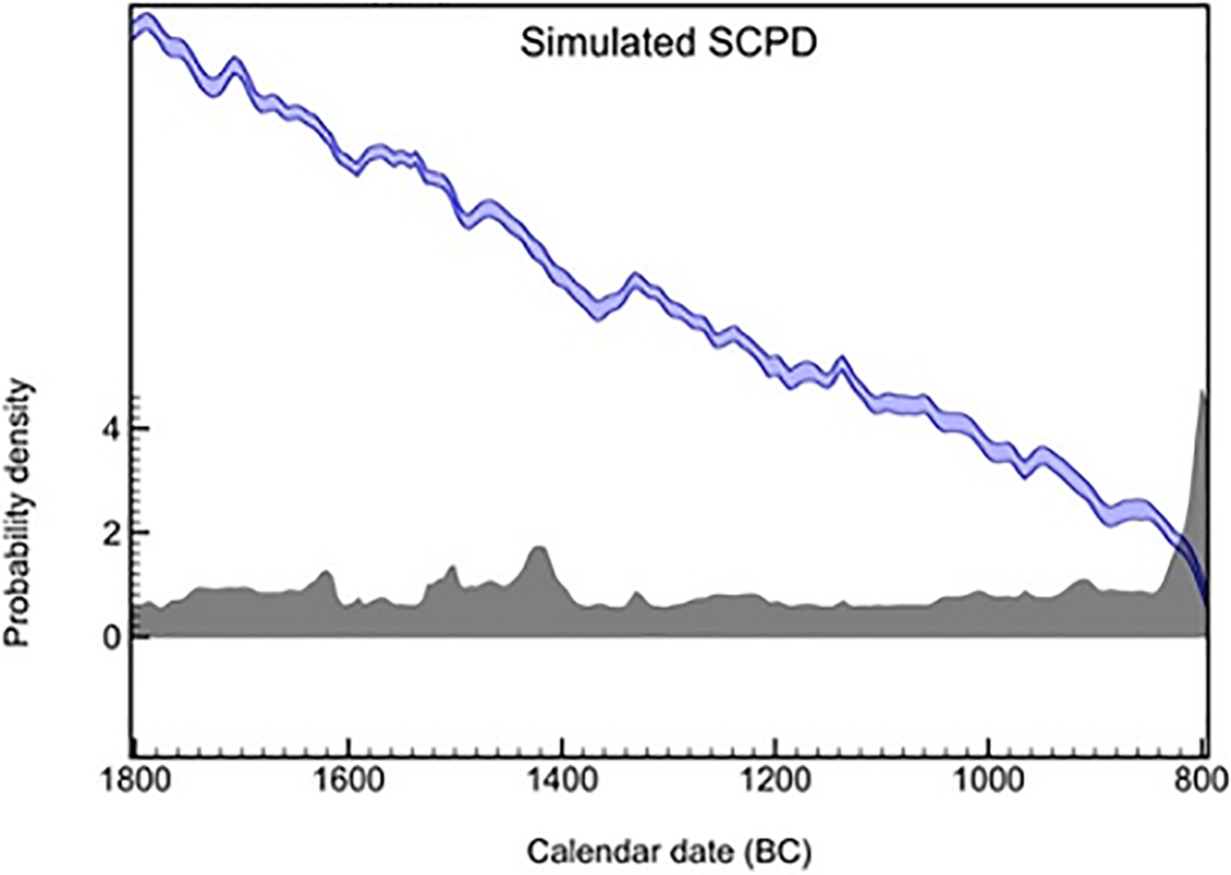
Simulated SCPD (dark grey, same number of dates at each temporal bin) compared with the IntCal13 calibration curve.

Since the effects of the calibration curve on radiocarbon estimates can alter the shape of the SCPD, we produced a simulated SCPD composed of uniformly distributed radiocarbon dates, under the assumption that the amount of dated archaeological contexts was the same for each year. The shape of the distribution does not change whether the amount of data per year is one or more than one. This was done in order to test a null hypothesis of no relationship between the observed SCPD and the effects of particular sections of the calibration curve, such as plateaus and calendar age steps [40,44,59]. A prominent peak in the simulated SCPD is visible at ca. 800 BC (Fig 4), corresponding to a steep calendar-age step between 860 and 700 BC in the calibration curve. A second peak, of considerably lesser magnitude, is visible at ca. 1420 BC, matching the calendar-age step in the intervals 1500–1380 BC. Beside the area around 800 BC, the simulated SCPD curve maintains a reasonably neutral trend, suggesting an overall limited influence of the calibration curve on our study time window.

### 2.2 Climatic reconstructions

In order to provide a simple environmental contextualization for the population trends within each region, we coupled the SCPD-based population data with local pollen-based climatic curves. The semi-quantitative reconstructions were performed using the Modern Analogue Technique (MAT) [60,61], focusing on two climatic variables: total summer (June, July, and August) precipitations and average summer temperature. The MAT relies on the assumption that pollen samples composed of a similar mixture of taxa are the byproduct of comparable vegetation assemblages. Therefore, given a modern pollen sample, the environmental conditions for its parent plant assemblage can be transferred to any fossil sample sharing a similar palynological composition [62]. The modern pollen samples used to build the calibration data set are derived from the European Modern Pollen Database (EMPD) [63].

We performed a basic quality filtering using the available EMPD metadata. Samples with known geolocation errors larger than 5 km were removed from the calibration data set. Following Mauri et al. [29], inaccurately georeferenced samples were also identified by comparing their elevation as recorded in the EMPD with elevation data for the same latitude and longitude extracted from a high-resolution Digital Elevation Model (DEM). We excluded all samples where the difference between EMPD and DEM data was higher than 250m [29]. Samples collected in riverine or estuarine contexts were removed too due to the presence of waterborne pollen potentially transported over long distances. Furthermore, we applied a minimum threshold of 400 pollen grains belonging to terrestrial species in order to select samples with rather stable taxa percentages (p. 165 in [64]). It was not possible to apply the same threshold to fossil data due to the limited number of sites available in some regions. Each modern pollen sample was then coupled with local present-day climatic parameters from the WorldClim dataset [65] using nearest-pixel extraction on the 30 arc-seconds resolution maps. The main source for the fossil archives used in the present paper is the European Pollen Database (EPD; http://europeanpollendatabase.net), which was locally integrated with additional sites. In the present study, we use the EPD version released on May 12, 2016. We constrained the selection of suitable fossil sites within each region by placing a 100 km-wide search window (130 km in the Po plain and Mediterranean areas due to limited data availability) around the location of each EUBAR site, then retaining only the EPD archives falling within its boundaries. No vertical constraint was applied on the Mediterranean region and the Po Plain due to the limited availability of fossil archives. We improved the coverage of these regions by including four additional sites: Banyoles [66,67] and Pla de l’Estany [68] in the Mediterranean area, and Castellaro Lagusello [15] and Forcello [69] on the Po Plain. We applied an upper elevation boundary of 1000 meters in the Massif Central area in order to exclude pollen archives that were located too far from the average elevation of the local EUBAR sites. The high availability of EPD sites in the Swiss plateau region allowed for a stricter constraint; here we selected only archives with the same vertical range of the local EUBAR sites (mean elevation ± 1σ). The complete list of the pollen archives used within each region is provided in Table 1. Semi-quantitative reconstructions were produced for each individual region with the exception of the Southern French coast and the Northeastern Iberian Peninsula. Here, the low number of suitable pollen records prompted us to produce a single set of climate curves – generically termed ‘Northwestern Mediterranean’– using records from both regions. The age-depth models for every site were obtained from Giesecke et al. [70] or were produced using the same methodology via Clam 2.2 [71]. Both modern and fossil pollen counts were converted to percentages based on the sum of terrestrial taxa, then aggregated into plant functional types (PFTs) [72]. We preferred the use of PFTs over the selection of indicator taxa, as PFTs reduce the need for taxa-specific modern analogues and can lessen the influence of anthropic disturbance on pollen assemblages [28,29,73]. Squared Chord Distance was preferred over other dissimilarity metrics due to its better performance in discriminating between vegetation types [74]. Climate reconstructions are based on the weighted average of the closest nine analogues. The number of relevant analogues was selected via leave-one-out cross-validation. The resistance of the model to spatial autocorrelation was tested via *h*-block cross-validation, where all samples within *h* kilometers of a test sample are omitted from analogue selection [75]. We opted for a value of *h* = 100 km for both climatic variables, since it should reasonably ensure that pollen source areas between the test sample and its potential analogues do not overlap (as inferable from, e.g., [76,77]), while at the same time preventing an excessive depopulation of the analogue pool. Both summer precipitations and temperature retained a satisfactory predictive power for *h* = 100 km, with r^2^ accounting for at least 50% of the variance and RMSEP lower than the standard deviations of the training sets (Table 2).

**Table 1:** Name and location of the pollen archives used for climatic reconstructions. Coordinates are expressed in decimal degrees (WGS84 reference system).

Region	Site name	Latitude	Longitude	elevation	n. in Fig 2
Swiss Plateau	Bibersee	47.20694	8.466667	429	1
	Durchenbergried	47.78333	8.983333	432	2
	Feuenried	47.75	8.916667	407	3
	Hornstaad/Bodensee	47.7	9.016667	385	4
	Lac de Clairvaux	46.565	5.749167	525	5
	Lac du Mont d'Orge	46.234	7.338167	640	6
	Lac d’Annecy	45.85667	6.172222	447	7
	Lobsigensee	47.03056	7.298056	514	8
	Montilier	46.935	7.123611	438	9
	Rotsee	47.07583	8.325833	428	10
Po Plain	Castellaro Lagusello	45.36926	10.63631	106	11
	Forcello	45.1114	10.83918	13	12
	Lago Piccolo di Avigliana	45.233	7.388333	356	13
Massif Central	La Taphanel	45.27444	2.679167	975	14
	Lac du Mont de Belier	45.33778	2.643056	860	15
	Lastioulles	45.38611	2.636111	854	16
	Peyrelevade	45.708333	2.383333	780	17
	Tourbière de Chabannes	45.64917	2.310556	800	18
Mediterranean	Banyoles	42.128975	2.752846	173	19
	Embouchac	43.56639	3.916667	1	20
	Etang d'Ouveillan	43.26667	3.00	6	21
	Pla de l`Estany	42.188697	2.531139	520	22
	Salada Pequeña	41.03333	-0.21667	357	23

**Table 2:** Performance summary for the h-block cross validation exercise. For each variable, we report the coefficient of determination (r2), the root of the mean squared error of the prediction (RMSEP) and the standard deviation of the observed climate variables.

	h-block cross-validation (h = 100 km)		Training data set
	r^2^	RMSEP	1σ
Summer precipitations	0.60	72.6	114.7
Summer temperatures	0.50	3.27	4.6

Temperature and precipitations are expressed as deviations from the mean of all reconstructions across the 1800–800 BC time-window within each region. The reconstructed values for each fossil site were averaged using overlapping windows with a span of 100 years and an increment of 50 years. A synthesis for each climatic parameter was then produced by fitting a LOESS curve to the combined values of every site in each region (smoothing span = 0.06). The age-depth models were used as a base to assess sample quality. Within each site, time windows located more than 1000 years away from the closest radiocarbon-dated point were excluded from the model. Furthermore, the average dating errors were employed as inverse weights in fitting the LOESS curve. All MAT-related analyses were performed using R [78] with packages rioja 0.9-9 [79] and fields 8.4-1 [80]. In addition to standard cross-validation techniques, we evaluated the reliability of these climatic reconstructions through a comparison with local, independent palaeoenvironmental proxies (see section 3).

## 3. Results

**Fig 5:**
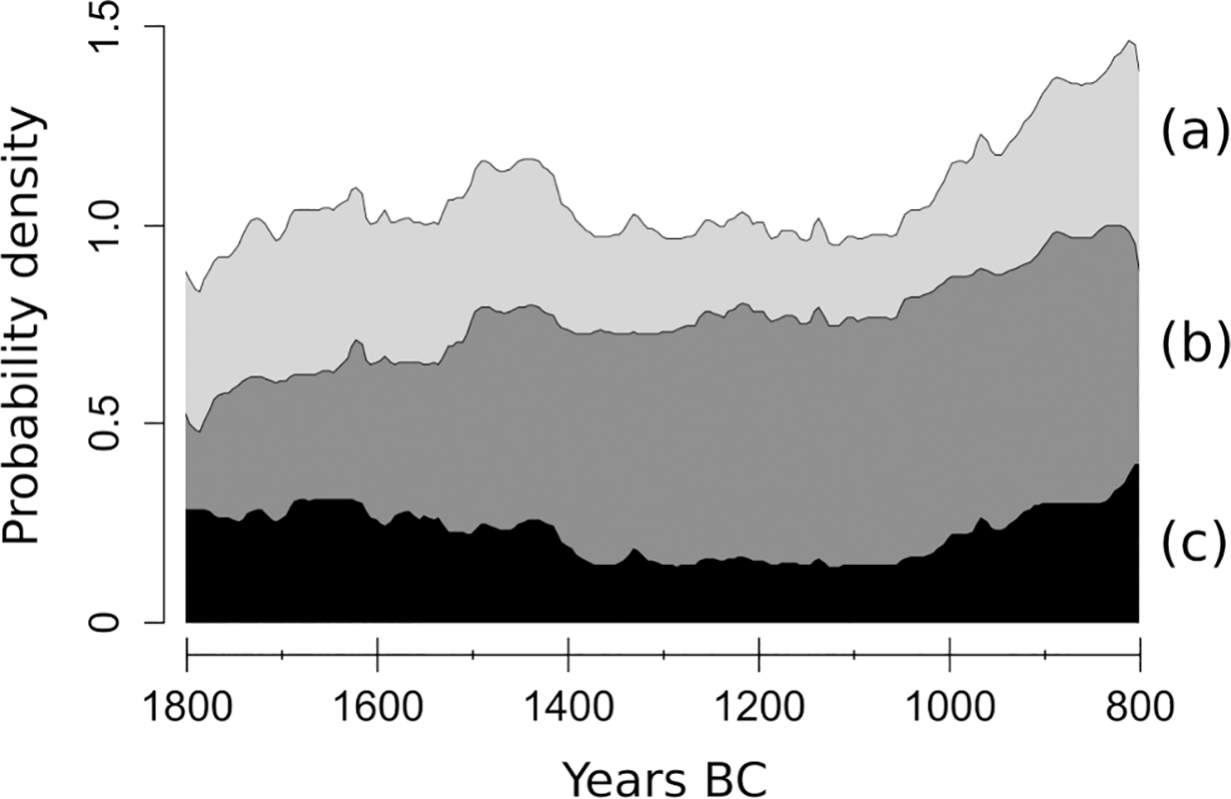
SCPDs of pre-screened radiocarbon dates from the EUBAR database, juxtaposed curves: (a) 1233 dates from the filtered dataset; (b) 852 ^14^C dates from settlements; (c) 283 ^14^C dates from funerary contexts (IntCal13 calibration curve).

At first glance, the general South European demographic reconstruction (Fig 5A) appears to be characterized by mild positive trends between ca. 1800 and 800 BC, interrupted by an apparent stagnation between ca. 1450 and 1050 BC.

In the period under investigation, a transition from the inhumation to the cremation rite occurs across several European regions [10,52,81–87]. To test the influence of this macroscopic shift in cultural practices on our reconstruction, we produce a second SCPD (Fig 5B) excluding dates from funerary contexts (Fig 5C). The resulting demographic curve is similar to Fig 5A -pointing to a limited influence of dates from cemeteries- and displays a more linear positive slope across the whole study window.

**Fig 6:**
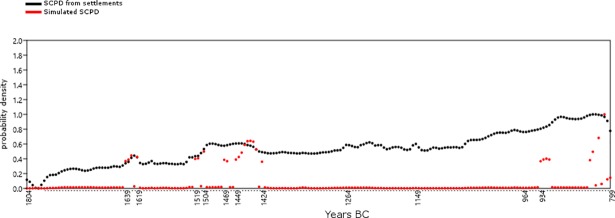
Probability density distributions of the SCPD with dates from settlements (black) and the simulated SCPD (red) after having been standardized (Software: PAST 3.18).

To evaluate further the effects of the calibration on the SCPD with dates from settlements, the empirical curve (Fig 5B) and the simulated SCPD (Fig 4) have been standardized in order to be compared (Fig 6).

The original data coincides mostly with the null-hypothesis of uniform distribution. The peak in the time-span 1639–1619 BC clearly matches a slope in the curve, and cannot be explained in terms of a sudden demographic change. In addition, the interval 1520–1430 BC coincides with two peaks of the calibration curve at approximately 1500 BC and 1430 BC. However, the changes in the calibration curve at 930 BC and 850 BC do not seem to leave any trace in the series.

**Fig 7:**
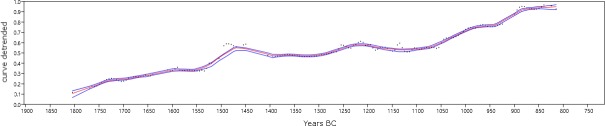
LOWESS function applied to SCPD data from settlements, the 95% confidence interval is marked by the blue lines (Software: PAST 3.18).

To minimize the effects of the greater or lesser slope of the IntCal13 calibration curve (Fig 4) on the empirical data, we applied a Locally Weighted Scatterplot Smoothing (LOWESS) [88,89] function that reduces spurious peaks and valleys (Fig 7). A 95% confidence interval has been generated starting from a simulated dataset equivalent in quantity to the number of unique events of the SCPD from settlements, and repeating the interpolation model 500 times. The confidence interval gathers most of these repetitions, each of them being a “possible demographic history”, given certain known sources of error and including the uncertainty in the relative population estimates over time [54].

**Fig 8:**
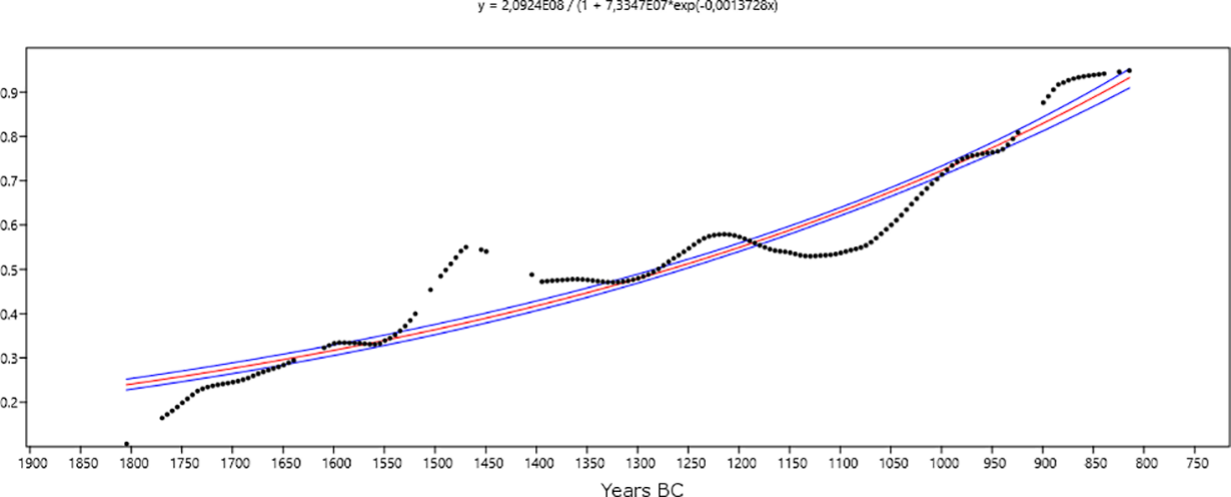
Standard logistic model fitted to the interpolated LOWESS function obtained from the SCPD data from settlements (Software: PAST 3.18).

In order to mathematically test the hypothesis of population growth within our study time window, we have adopted a statistical approach in which the empirically verified variation is compared to the values that would reflect the null hypothesis, i.e. the absence of population growth. In fact, only if the observed variation proves to be significantly different from the null hypothesis we can accept the existence of a certain pattern of change. In other words, we can stress the existence of a statistically significant demographic growth (or decrease) if the probability at a given point of the curve exceeds the value expected if the null hypothesis were correct. As a consequence, we have curve-fitted the values predicted by the interpolated LOWESS function obtained from SCPD data from settlements, since it minimizes the effects of the calibration process; the null hypothesis to be tested is the neutral growth of the population, expressed in terms of a standard logistic model [90,91] (Fig 8).

**Fig 9:**
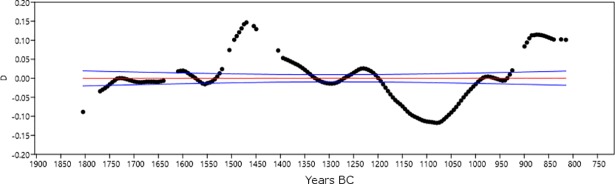
Linear trend of the interpolated LOWESS function obtained from the SCPD data from settlements (Software: PAST 3.18).

To make the interpretation of the results easier, the linear trend in the transformed empirical series has been extracted and the linear model has been used as a baseline to detect peaks and valleys in the series (Fig 9).

The probability peak around 1450 BC still persists in the transformed series and it is clearly higher than it would be expected if the null hypothesis were true, indicating one or more episodes of population growth in the time span 1550–1450 BC. The population decrease after around 1470 corresponds to a period of variations in solar activity as attested by the calendar-age step in the intervals 1500-1380 BC, as mentioned above. Therefore, it is possible to suppose that there is a link between the two phenomena. More significant seems to be the possible population increase around 1250–1200 BC and the decrease in the probability of dating settlements between 1200 and 1050 BC. The possible population recovery after that time-span allows merely to reach the level of neutral growth, which would be surpassed only after 900 BC.

For a better understanding of these phenomena we need to analyze the demographic behavior of the five study regions (Swiss Plateau, the Po Plain, the Massif Central, the Southern French coast, and the Northeastern Iberian Peninsula), which is characterized by various and pronounced episodes of population growth or decline.

### 3.1 Swiss Plateau

**Fig 10:**
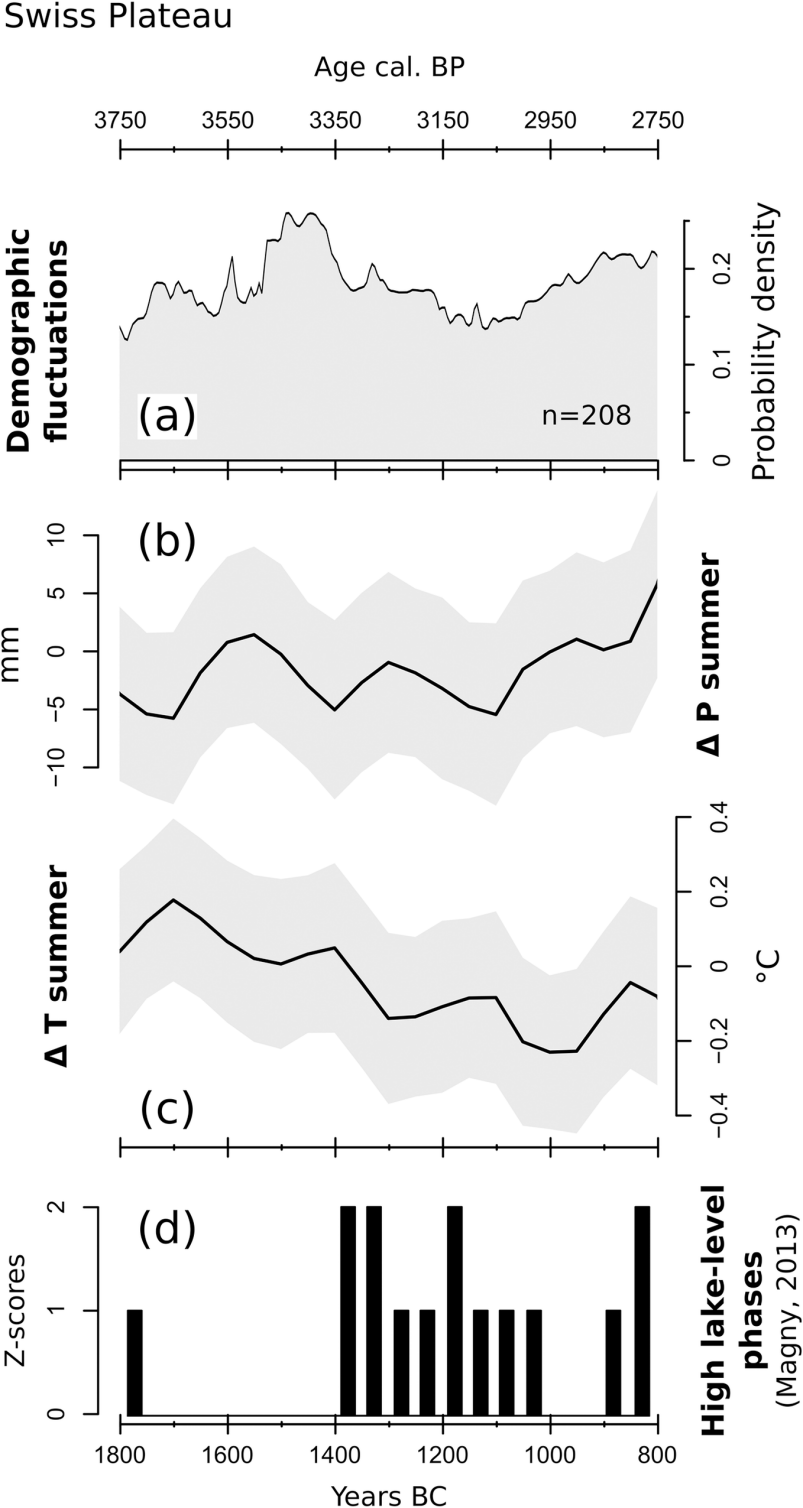
(a) SCPD of 208 ^14^C dates originating from sites located on the Swiss Plateau; (b) LOESS model of reconstructed summer precipitations; (c) LOESS model of reconstructed summer temperatures; (d) High lake level z-scores digitized from Magny (2013). The shaded areas in (b) and (c) outline the 95% confidence interval of each LOESS model.

The Swiss Plateau SCPD (208 radiocarbon measurements collected from 81 archaeological sites) shows two phases characterized by a general positive trend, extending from the beginning of our study time window to around ca. 1500–1450 BC and from 1100 BC to its end, respectively (Fig 10A). The interposed demographic decline (around 1450–1100 BC) shows a positive correlation with the abandonment of lakeside dwellings in the region [9,92–94]. Dwelling abandonment phases in the area appear to correspond with lake-level changes, which in turn have been linked to unfavorable climatic fluctuations [92]. The lake-level model presented by Magny [95] describes a high lake-level (HLL) phase lasting from ca. 1400 to 1150 BC (Fig 10D), possibly linked to the establishment of prevalently wetter and cooler conditions [92].

Magny’s HLL phases are identified by combining the chronological record of HLL events from multiple sedimentary archives. In the resulting histogram, non-zero Z-scores contribute to define regionally prominent HLL phases [96]. An extended negative trend in the SCPD curve appears to support a connection between population decline and the 1440–1150 BC HLL phase. This matching behavior finds only a partial parallel in the pollen-based climatic curves (Fig 10B and 10C). At a mere trend level, the summer temperature curve does indeed reach its lowest values between ca. 1400 and 950 BC. Furthermore, Magny [92] points out that the sedimentary record of Lake Clairvaux (Jura, France) suggests a climatic downturn in the region as early as 1650–1600 BC, i.e. when temperatures begin to decline in our model. On the other hand, the precipitation curve is characterized by a see-saw pattern that describes an overall neutral trend and displays rather limited similarities with HLL Z-scores. Still, it should be noted that the magnitude of the fluctuations in both pollen-based curves remains rather modest, possibly implying a limited sensitivity of the local plant communities to any climatic factor affecting lake levels.

### 3.2 Po Plain

**Fig 11:**
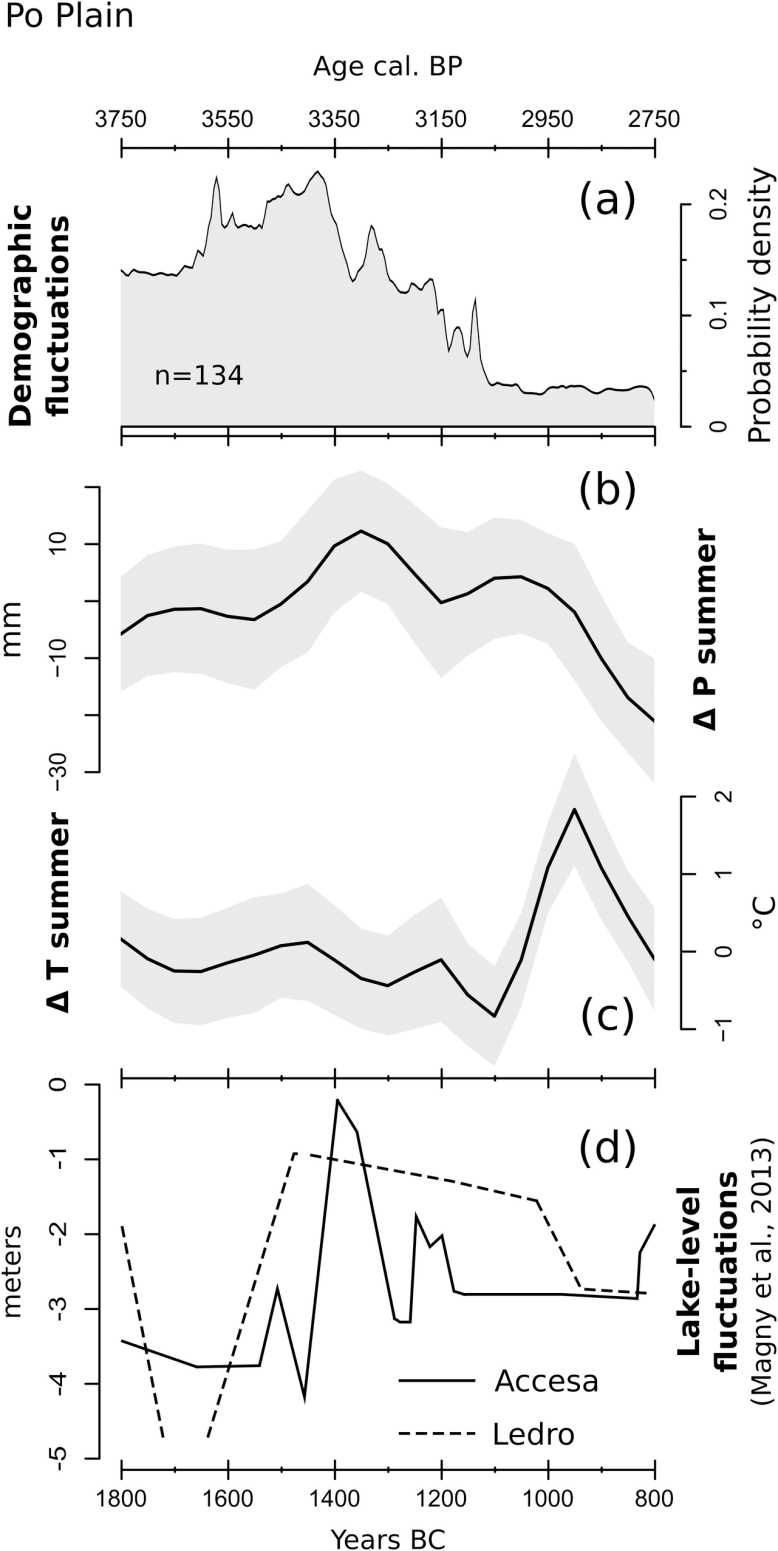
(a) SCPD of 134 ^14^C dates originating from sites located in the Po Plain; (b) LOESS model of reconstructed summer precipitations; (c) LOESS model of reconstructed summer temperatures; (d) Water level fluctuation for Lake Ledro and Lake Accesa, digitized from Magny et al. (2013). The shaded areas in (b) and (c) outline the 95% confidence interval of each LOESS model.

The SCPD for the Po Plain is based on 134 radiocarbon dates from 46 archaeological sites (Fig 11A). The resulting demographic curve is characterized by a distinct tripartite behavior, displaying notable similarities to both the local archaeological narrative and the climatic reconstructions. The first portion of the curve shows a population increase that eventually peaks at ca. 1500–1450, and is then followed by a severe decline lasting until ca. 1100 BC. The remaining portion of the curve shows low values, reflecting both a sparse human presence and extended demographic stagnation.

During the local transition from the Middle Bronze Age I to the Middle Bronze Age II, ca. 1500 BC according to de Marinis [97], wetland settlements in the Garda Lake region tended to move to higher grounds and dwellings in the alluvial plain were surrounded by earthen ramparts and ditches, suggesting a general increase in humidity [98].

Consistently with this interpretation, the precipitation curve (Fig 11B) peaks at 1400 BC, pointing to a higher precipitation phase that is also supported by water level reconstructions for Lake Ledro and Lake Accesa (Fig 11D). Both these lakes, located in the Southern Alps and in Central Italy respectively, show a remarkably similar trend with peaks in water depth around ca. 1500–1400 BC [99]. The visible decline in population after ca. 1400 BC appears then to match a following gradual transition to drier conditions (Fig 11B and 11C), in agreement with the gradual drying up of wetland areas and decline of groundwater levels inferred from independent palaeoecological and archaeological data [14,15,100,101].

### 3.3 Massif central

**Fig 12:**
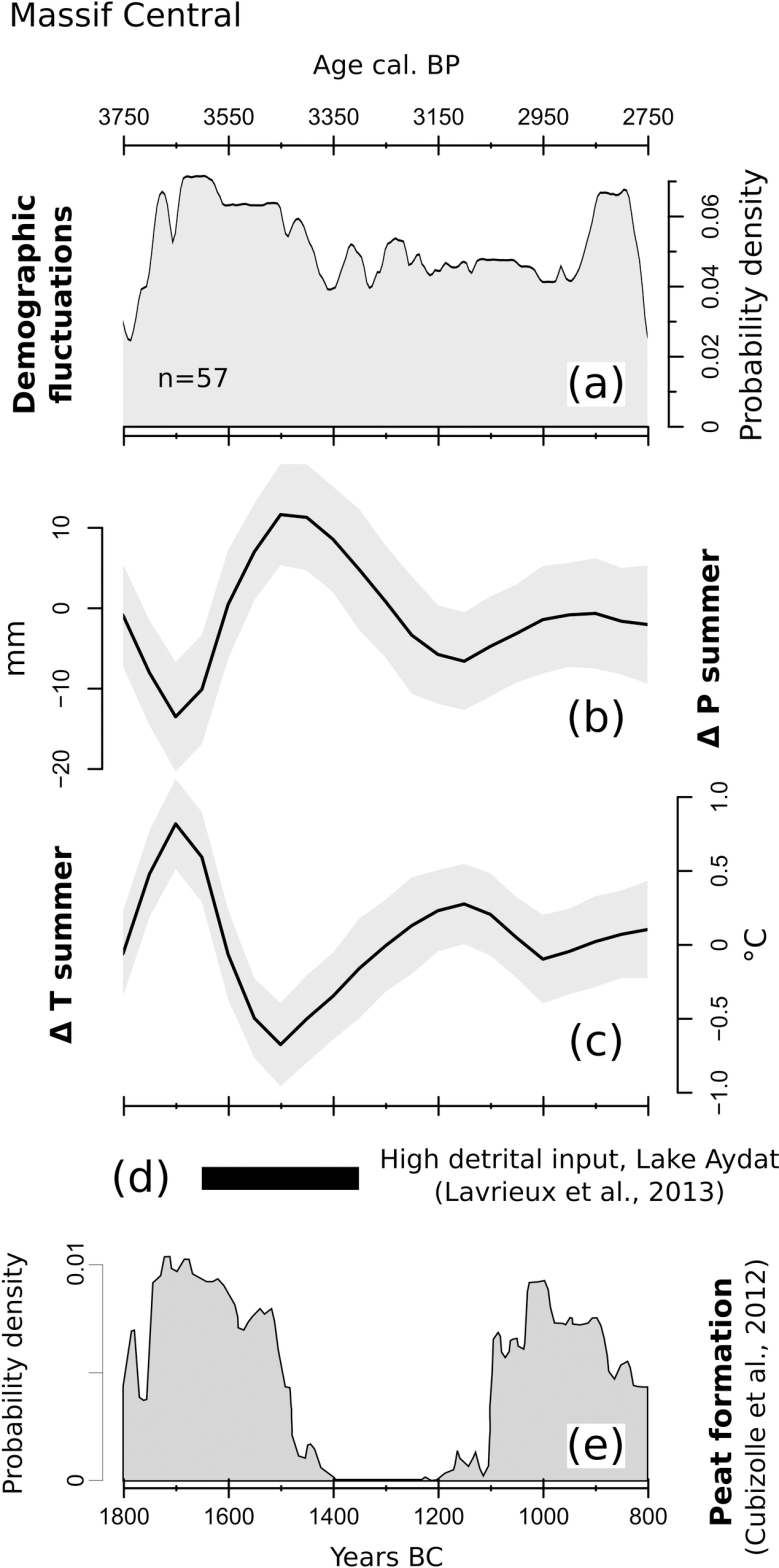
(a) SCPD of 57 ^14^C dates originating from sites located in the Massif Central; (b) LOESS model of reconstructed summer precipitations; (c) LOESS model of reconstructed summer temperatures; (d) Duration of high detrital phase in Lake Aydat (Lavrieux et al., 2013); (e) SCDP-based peat initiation events digitized from Cubizolle et al. (2012). The shaded areas in (b) and (c) outline the 95% confidence interval of each LOESS model.

The SCPD for the Massif Central region is based on 57 dates from 33 archaeological sites (Fig 12A).

A visual comparison highlights few similarities between the demography and climate, although a cautious interpretation remains necessary due to the limited number of radiocarbon dates composing the SCPD curve. The sharp population increase observed between 1800–1700 BC coincides with a transition to warmer/drier conditions (Fig 12B and 12C), and the subsequent general decline (ca. 1700–1400 BC) appears to occur in correspondence with a wet and cold shift. The following climatic fluctuations -most notably a shift towards drier and warmer conditions after ca. 1500 BC- are not met with equally visible demographic changes. On the contrary, the SCPD curve remains rather stable across the most of the study window, pointing to an overall prevailing and long-lasting neutral demographic trend.

The notable 1700–1500 BC climatic deterioration finds a first parallel in the high detrital input phase recorded in nearby Lake Aydat between ca. 1650 and 1350 BC [102] (Fig 12D), arguably linking wetter/cooler conditions to long-term soil erosion. A predominant climatic trigger for this sediment discharge was suggested especially after ca. 1550 BC [103], but local anthropic land use appears to be rather limited throughout the whole Bronze Age (e.g., rare occurrences of cropland/pastoral pollen indicators and coprophilous fungi). The Lake Aydat record is interrupted between ca. 1230 BC and 180 AD due to sediment mixing, thus not covering the minor shift to wetter and cooler conditions visible from 1150–1000 BC in the pollen-based curves. Cubizolle et al.[104] offer an additional insight on local landscape development through an SCPD-based record of peat formation events for the Eastern Massif Central (Fig 12E). Cubizolle et al. [104] suggest that anthropic land use was a driving mechanism behind peat initiation, partly basing their hypothesis on climatic reconstructions external to the study area (i.e. [95,105]). A visual comparison between peat formation and demographic trends reveals limited similarities, which nonetheless should not be exceedingly stressed due to the limited number of radiocarbon dates involved. Still, it is worth noting that a visible gap in the peat formation curve (ca. 1400-1200 BC) occurs together with a transition into warmer and drier conditions, and comes to an end after a minor cold and humid shift. These similarities between peat formation events and climate behavior might depend on known wetland expansion trends occurring under cool and moist conditions [106], which would ultimately support the validity of our reconstructions and possibly point to a prevailing climatic control on local wetland dynamics during the Bronze Age.

### 3.4 NW-Mediterranean

**Fig 13:**
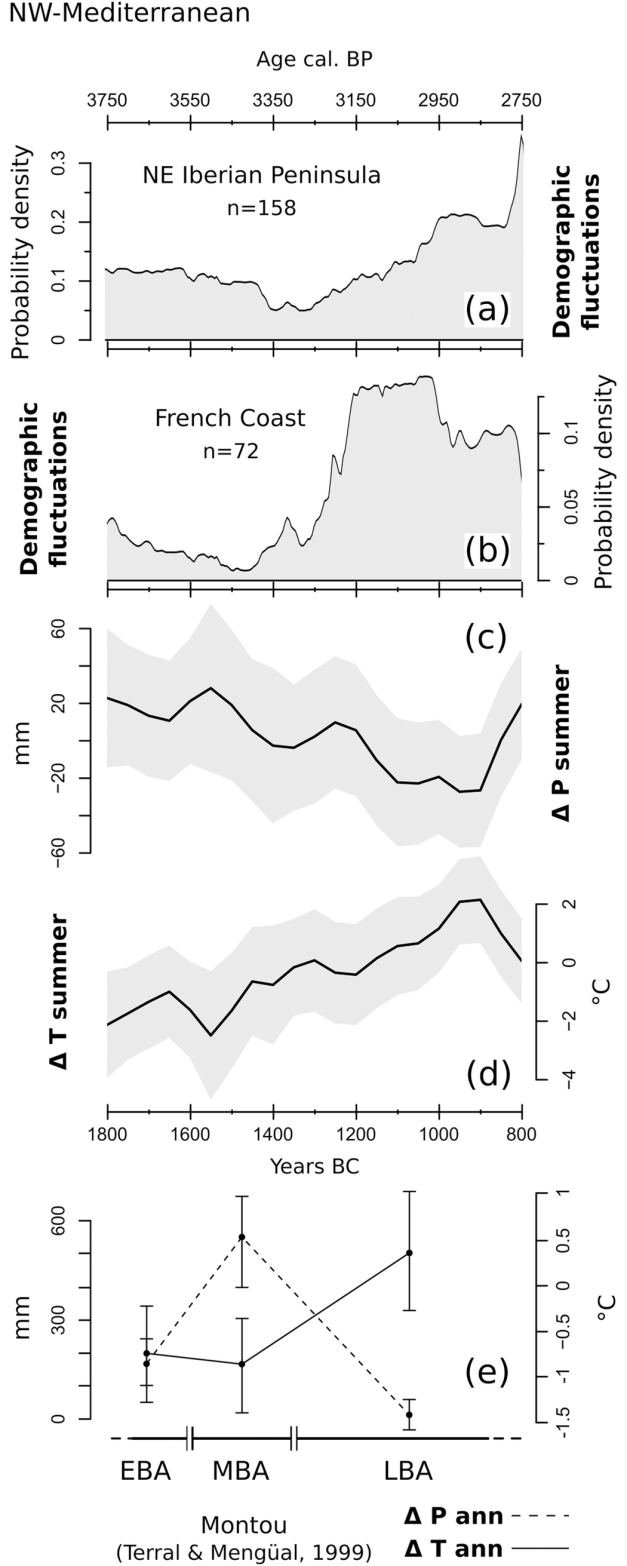
(a) SCPD of 158 ^14^C dates originating from sites located on the northeast Iberian Peninsula; (b) SCPD of 72 ^14^C dates originating from sites located on the Southern French coast; (c) LOESS model of reconstructed summer precipitations; (d) LOESS model of reconstructed summer temperatures; (e) Annual temperature (ΔTann) and precipitation (ΔPann) reconstructions from the site of Montou (SW-France), digitized from Terral and Mengual (1999). The shaded areas in (b) and (c) outline the 95% confidence interval of each LOESS model.

The SCPD of the two remaining regions, the Southern French coast and the Northeastern Iberian Peninsula, are discussed together due to their similarities (Fig 13A and 13B). As mentioned in section 2.2, a single set of Northwestern Mediterranean temperature and precipitation curves is produced using pollen archives from both areas (Fig 13C and 13D). The population curve for Southern France (Fig 13B) is based on 72 dates from 19 archaeological sites located in a buffer zone of 40 km from the Mediterranean coastline. The SCPD curve begins with a negative trend, reaching its lowest point at around 1450 BC. The subsequent population recovery follows an exponential trajectory until ca. 1200 BC, and is then followed by an extended period of stagnation/decline. The Northeastern Iberian dataset is composed of 158 radiocarbon dates from 74 archaeological sites (Fig 13A). The SCPD curve shows visible similarities with the neighboring Southern French coast, but with a lag of ca. 100–200 years. The lowermost values occur between 1400 and 1300 BC, following a rather stable/mildly declining phase. The curve acquires a positive trend until ca. 1000 BC. The remaining portion is then characterized by stable conditions that turn into rapid growth towards the very end of our temporal window.

The temperature and precipitation curves display a specular behavior pointing toward an ongoing transition into warmer and drier conditions. A comparable situation, with overall declining annual precipitations and rising annual temperatures between the Middle and Late Bronze Age, was found at the coastal site of Montou (Southern France) based on variations in olive wood anatomy [107] (Fig 13E). These general trends fit well with the gradual aridification process of the Western Mediterranean region, leading from a wetter-than-present mid-Holocene to the current Mediterranean climate [108,109].

## 4. Discussion

In the present paper we offer an SCPD-based reconstruction of population dynamics across Central-Southern Europe and within different smaller regions in order to check for any similarity/difference between neighboring areas and evaluate them against existing narratives. As a further exercise, we compare these SCPD-based curves with local, semi-quantitative climatic reconstructions. This comparison aims at identifying the presence or absence of macroscopic matching or specular patterns, in order to evaluate the presence of potential phases of climatic control on human activities. We interpret our findings taking into account the available local archaeological and palaeoenvironmental evidence. Nonetheless, we recognize that any schematic comparison can hardly unravel the complex mechanisms behind human population dynamics. Taking into account the potential uncertainties affecting the reconstructions (both demographic and climatic; e.g. combinations of dating accuracy and limited data availability) we opted to avoid a statistically supported (cross-)correlation analysis of our results. We considered a visual comparison between curves appropriate for the available data and the scope of the paper.

Regarding the possible issues on sample size for regional analyses it is essential to highlight that the one-millennium temporal window considered in the present study is much shorter than the ones commonly adopted in the available literature. The approximate data density (dates/year) for the regional analyses ranges between 0,06 dates/year (Massif Central) and 0,21 dates/year (Swiss Plateau). The geographic framework of the paper spans from the ca. 15000 km^2^ of the “Swiss Plateau” region to the ca. 670000 km^2^ of the whole area. In terms of spatial density, the lowest value is reached by the Massif Central region with 0,001 dates/km^2^ whilst the highest value is recorded in the Swiss Plateau 0,14 dates/Km^2^. These values are comparable with those of already published research involving the SCPD method (e.g. literature review in [110]).

Despite a relatively high regional data density per year, it remains important to take into account the limited absolute number of radiocarbon dates available after screening and combination. Instead of the very general recommendations for big datasets [40], we have opted for thresholds based on the temporal range –in this case, 1000 years- and the minimum date for approximating the underlying Poisson distribution with a Gaussian distribution. According to that criterion, the general demographic trend for each region is within such limits.

On a macroscale, the European demographic curves (Figs 8 and 9) display episodes of distinct population growth (between ca. 1550–1450 BC and after ca. 1050 BC) interrupted by a rather steady negative trend (ca. 1450–1050 BC). This behavior reflects a combination of different and more dynamic regional trajectories.

On the Swiss Plateau, positive SCPD trends (1800–1450 BC; 1050–800 BC) appear to fit well with population recovery periods following phases of settlement discontinuity (2400–2100 BC; 1500–1100 BC; [111]). The reasons behind the population decline from 1500–1100 BC are still debated. The wide spectrum of cultivated crops might have proven to have been an efficient protection against harvest failures, a hypothesis that is supported by the not-increasing role of hunting activities during unfavorable climatic conditions [112]. Socioeconomic factors might have played a visible role only at a later stage, between the Late Bronze Age and the Early Iron Age, when a significant reduction in the quantity of swords in archaeological contexts could suggest a declining importance of Swiss trade nodes within inter-regional exchange routes [113,114]. A combination of cooler conditions and rising lake levels is currently regarded as an important contributing factor behind human displacement events [114]. Our reconstructions point to only a minor shift towards cooler and wetter conditions, yet they suggest that climate deterioration might have occurred in the area since the end of the 17th century BC. Conversely, population decline occurred only two centuries later according to the archaeological record. This late reaction might point to a high degree of resilience among the local communities to adverse climate events, yet the effective magnitude of this unfavorable climatic trend remains to be more precisely quantified. Only a further harshening of (potentially already) unfavorable conditions might have then resulted in settlement relocation and population decline (namely, the ca. 1500–1100 BC lake-dwelling hiatus), but not in widespread regional abandonment. Even during the coldest phase within our study time window, the area was never completely depopulated, as suggested by the relative abundance of radiocarbon-dated archaeological contexts.

The SCPD curve on the Po Plain exhibits changes that are more dramatic. The period between ca. 1650 and 1350 BC marks the maximum development of Bronze Age settlements between the Alps and the Apennines [11,12,115] corresponding to the population peak observed in the SCPD curve. These overall favorable conditions for population growth are possibly linked to a transition from slash-and-burn forms of agriculture to more productive irrigated croplands [116], even though the direction of causality does not appear to be solvable with the available data. The stable negative SCPD trend after ca. 1400 BC is somewhat in contrast with the traditional interpretation of archaeological data, which points to a sudden abandonment phase only after 1200 BC [117]. A possible explanation behind this disparity lies in the reorganization of the settlement system in the Terramare area (Eastern Po Plain) after ca. 1450 BC, characterized by population agglomeration in larger settlements -and abandonment of smaller ones- rather than by the establishment of new villages [12]. Nonetheless, in agreement with the traditional interpretation, our model shows an extended period of depopulation after ca. 1150 BC in connection with the establishment of warmer and then more arid conditions. Significantly, the end of the Terramare culture around 1150 BC might have been triggered by a water crisis, as suggested by the lowering of the local water table over time [14]. This shift towards arid conditions arguably affected an environment already deteriorated by landscape over-exploitation and extensive deforestation [13–15,118,119]. The resulting widespread societal collapse affected the entire Southern Po Plain, which became largely void of settlements until the beginning of the Iron Age [11,117,120–122].

The Massif Central area is the least represented in terms of number of sites. Assuming an adequate coverage and representativeness of the radiocarbon dataset, the low number of data-points does suggest a rather diluted human presence within our time window. This situation might primarily reflect difficulties in establishing a durable and extensive occupation in an area characterized by a mountainous landscape [123]. Consistently, cropland indicators in pollen diagrams are limited to sparse pollen grains, possibly pointing to subsistence strategies more focused on livestock [124,125]. During the second and the beginning of the first millennium BC, interregional cultural contacts are attested by the presence of specific pottery typologies, such as vessels with handles *ad ascia* attributed to Italic influences transmitted through the Languedoc and Provence [126,127]. The main trade route in Bronze Age Southern France followed the course of the Rhone River, between the western Alps and the Massif Central, placing the latter in a peripheral but increasingly dynamic situation between the Mediterranean coast and Central France. Despite the patchiness of the radiocarbon record, the fluctuations in the SCPD curve may indeed reflect major regional cultural dynamics. The first peak in human activities (around ca. 1700 BC) occurs during the Early Bronze Age, when the dynamism of the local agro-pastoral communities is testified by regional and interregional cultural contacts, as well as by signs of emerging social stratification [126,128]. A change occurs with the transition into the Middle Bronze Age (after ca. 1600 BC), when the archaeological record points to a general abandonment of lowland sites and the use of low and middle mountainous belts [123,128]. In this context, the contemporaneous transition to colder and wetter conditions, coupled with the sedimentary discharge visible in Lake Aydat, might point to population displacement as a response to increased hydrogeological instability. Nevertheless, both the SCPD curve and the persistence of long-distance contacts in the archaeological record suggest a reorganization of the settlement system towards increased mobility rather than a complete abandonment of the area [128]. A radical landscape transformation is recorded in the region only since the transition into the Iron Age (ca. after 800 BC), with an increasing presence of funeral mounds, the establishment of *oppida* (hilltop sites), and the first large-scale deforestation events [128].

The NW Mediterranean presents a situation clearly distinct from the other regions. The visibly similar trends between the French coast and the NE Iberian Peninsula reflect the commonalities shared by these two regions. Trans-Pyrenean and maritime fluxes of people spread innovations from Southern France to the northeastern Iberian Peninsula, in particular from the Middle Bronze Age onwards. Evidence for these cultural connections can be found in the adoption of specific pottery typologies, such as vessels with handles with vertical expansion (*asas de apéndice de botón*), fluted pottery (*cerámica acanalada*), and the arrival of cremation burials [82–87,129,130]. The NE Iberian area probably played a modest role during the Early/Late Bronze Age when compared with more structured cultural centers located in the southeastern and central parts of the peninsula [131]. This peripheral situation appears to be visible in the stagnating SCPD values from ca. 1800–1450 BC, partly overlapping with the widespread abandonment phase that follows the southeastern Argaric collapse (ca. 1550 BC; [132]). Internal forces or a subsistence crisis are mentioned as leading causes for this sudden cultural shift [131,133]. Still, potential connections between these events and the ca. 1400–1300 BC SCPD minimum in NE Iberia remain unclear. The demographic growth registered after this phase matches rather precisely the local emergence of the cremation ritual, possibly suggesting population fluxes from continental Europe [83,84,131]. The simultaneous transition towards a more arid climate does not appear to hinder population growth. Similarly, positive demographic trends in Southern Iberia in co-occurrence with increasingly arid conditions have already been described between ca. 3550 and 2550 BC [134]. Notably, drought resistant barley is the dominant cereal during the entire Bronze Age in Mediterranean Spain [135].

Of particular interest is the demographic increase registered along the French coast between 1350 and 1200 BC. This phase of demographic growth appears to predate the major cultural phenomenon in the area, i.e. the expansion of the Mailhacien culture around 900 BC [136–138], which in turn occurs during a period of relative stability in the SCPD curve. In this regard, the apparent mismatch between the SCPD and the archeological data might depend – at least partially – on cultural differences between the western and the eastern French coast (Gascó, 2000, 2011; Janin, 2000; Mordant, 2013; Vital, 1999, 2001; Vital et al., 2012). A more appropriate intra-regional discussion is currently prevented by the limited number of available radiocarbon dates, which led us to group the French Riviera, Provence and Languedoc-Roussillon under a single SCPD despite their heterogeneous archeological dynamics.

## 5. Conclusions

In the present paper, we use a dataset of archaeological radiocarbon dates to reconstruct demographic trends in Central-Southern Europe between 1800 and 800 BC. On a macroscale, a positive demographic trend is visible until ca. 1450 BC, and is then followed by a phase of population decline lasting until ca. 1050 BC. Until ca. 1050 BC, the macroscale population dynamics appear to be mostly determined by circum-alpine trends. At the beginning of the 16th century BC there was an expansion of the Terramare settlements, in which the entire populated area in the Po Plain tripled in size [139]. Similarly, in the French Jura Mountains it is attested a period of population growth around 1500 BC [140]. Significantly, northern Italy, eastern France, the ore-rich Alpine area were economically the most dynamic regions after 1600 BC in Europe, with clear evidence of a socio-economic growth [141]. The population decrease after ca. 1470 BC coincides then with the crisis of the lake-dwelling settlement system in the Circum-Alpine region [9,93,111,142]. Similarly, the demographic contraction between 1200 and 1050 BC takes place in a time-span defined by the collapse of the pile-dwelling/Terramare culture around 1150 BC [13–15]. A renewed episode of macroscale demographic growth is visible after ca. 1050 BC, likely reflecting both the population peaks recorded along the NW Mediterranean coast and the population recovery trend visible in the Swiss Plateau. Notably, the prominent NW Mediterranean positive trends occur in connection with the local adoption of the cremation ritual, and might imply a demographic influx from central Europe.

A potential relation between population trends and climate was visually evaluated by comparing the SCPD curves with semi-quantitative summer temperatures and precipitation curves reconstructed from pollen sequences specific for each region. Climate appears to play a non-dismissible role on the Po Plain, where widespread settlement abandonment occurs in connection with increasingly arid conditions. While independent archaeological and palaeoenvironmental proxies support this connection, it remains unfeasible to ascertain whether climate represents the main forcing factor leading to the collapse of local Bronze Age societies, or if its effects were exacerbated by additional and cumulating factors, such as an inferred landscape overexploitation. Communities on the Swiss Plateau appear to show a higher degree of resilience to climatic fluctuations, with settlement collapse occurring only during the coolest interval in the temperature curve, after centuries of increasingly cooler conditions. A population drop in the Massif Central occurs in connection with a particularly pronounced cold and wet phase (ca. 1700–1500 BC). Apart from this event, the local communities appear to be largely unaffected by other climatic shifts. The predominant stagnation emerging from the Massif Central SCPD curve seems to reflect a long-term stability rather uncommon among our reconstructions, presumably connected to low demographic density and high mobility deriving from a livestock-based subsistence.
